# A landmark-based approach to locate symptom-specific transcranial magnetic stimulation targets of depression

**DOI:** 10.3389/fpsyg.2022.919944

**Published:** 2022-09-02

**Authors:** Rongrong Du, Qian Zhou, Tianzheng Hu, Jinmei Sun, Qiang Hua, Yingru Wang, Yuanyuan Zhang, Kongliang He, Yanghua Tian, Gong-Jun Ji, Kai Wang

**Affiliations:** ^1^Department of Neurology, The First Affiliated Hospital of Anhui Medical University, Hefei, China; ^2^The School of Mental Health and Psychological Sciences, Anhui Medical University, Hefei, China; ^3^Anhui Province Key Laboratory of Cognition and Neuropsychiatric Disorders, Hefei, China; ^4^Collaborative Innovation Centre of Neuropsychiatric Disorder and Mental Health, Hefei, China; ^5^ANDE College, University of Architecture and Technology, Xi’an, China; ^6^Institute of Artificial Intelligence, Hefei Comprehensive National Science Center, Hefei, China; ^7^Department of Neurology, The Second Affiliated Hospital of Anhui Medical University, Hefei, China

**Keywords:** TMS, coil positioning, target accuracy, depression, MRI

## Abstract

**Objective:**

Two subregions of the dorsolateral prefrontal cortex have been identified as effective repetitive transcranial magnetic stimulation (rTMS) targets for the “anxiosomatic” and “dysphoric” symptoms, respectively. We aimed to develop a convenient approach to locate these targets on the scalp.

**Materials and methods:**

In a discovery experiment, the two personalized targets were precisely identified on 24 subjects using a neuronavigation system. Then, a localized approach was developed based on individual scalp landmarks. This “landmark-based approach” was replicated and validated in an independent cohort (*N* = 25). Reliability of the approach was tested by calculating the correlation of both the inter-rater and intra-rater results. Validity was tested by comparing the mean distance between the personalized and landmark-based targets to the TMS spatial resolution (i.e., 5 mm). We further conducted a total of 24 sham rTMS sessions to estimate the misplacement between the coil center and target during a 10-min stimulation without neuronavigation.

**Results:**

The parameters of the “landmark-based approach” in the discovery experiment were replicated well in an independent cohort. Using discovery parameters, we successfully identified the symptom-specific targets in the independent cohort. Specifically, the mean distance between the personalized and landmark-based targets on the cortex was not significantly larger than 5 mm. However, the personalized and landmark-based targets distance exceeded 5 mm in more than 50% of subjects. During the 10-min sham rTMS session, the average coil misplacement was significantly larger than 5 mm.

**Conclusion:**

The “landmark-based approach” can conveniently and reliably locate the two symptom-specific targets at group level. However, the accuracy was highly varied at individual level and further improvement is needed.

## Introduction

Repetitive transcranial magnetic stimulation (rTMS) is an established treatment for patients with Major Depressive Disorder (MDD) who have failed to respond to one or more medication trials (Perera et al., 2016). The dorsolateral prefrontal cortex (DLPFC) is the most commonly used TMS stimulation area for depression.

There are several commonly used methods to define the therapeutic target on the left DLPFC, in which the scalp measurement approach is the most widely used method. For instance, the “5 cm method” defined the stimulation point as being 5 cm anterior to the motor cortical hotspot (George et al., 1995; Pascual-Leone et al., 1996). In some studies, the stimulation site has been modified to 5.5 or 6 cm. An alternative approach has involved localizing stimulation to the F3 electroencephalogram location based on the standard 10–20 method (Herwig et al., 2003). This method takes head size into account, by using specialized elastic caps, or *via* numerical algorithms such as “BeamF3” (Beam et al., 2009). To improve the accuracy of target localization, many studies adopted individual brain imaging to locate individualized target. These studies combined the accuracy of the brain imaging and the simplicity of scalp measurement. For instance, the triangulation-based MRI-guided method by Andoh et al. (2009) can locate NIBS target with only individual anatomical scan and the GeodesicSlicer tool by Briend et al. (2020) that helps to locate stimulation site with individual brain anatomical image and then mark it on the scalp according to 10–20 system EEG electrode positioning.

Many studies adopted individual MRI to navigate the stimulation online. These neuroimaging studies have been synthesized to establish a consistent or common stimulation target by meta-analysis (Fitzgerald et al., 2006, 2008). Emerging evidence suggests that separate symptom clusters might respond to the stimulation of different brain circuits (Downar and Daskalakis, 2013; Drysdale et al., 2017; Downar, 2019). In MDD patients, Siddiqi et al. (2020) explored the relationship between the target network and multiple psychological symptoms. They found two discrete clusters of symptoms - One cluster named the “dysphoric” symptoms, included symptoms such as sadness, decreased interest, and suicidality, and another cluster named the “anxiosomatic” symptoms, included symptoms such as irritability, sexual disinterest, and insomnia. Correspondingly, functional connectome analysis identified two spatially discrete targets in the left DLPFC. The “dysphoric” target is part of a brain network variably referred to as the ventral attention, salience, or cingulo-opercular network (Thomas Yeo et al., 2011). The peak dysphoric target was at MNI coordinates [−32, 44, 34], close to the “anti-subgenual” target used in recent TMS trials (Blumberger et al., 2018; Williams et al., 2018), whereas the peak anxiosomatic target was at MNI coordinates [−37, 22, 54], which is part of the default mode network (Thomas Yeo et al., 2011).

According to the coordinates provided by Siddiqi et al., researchers could easily identify the symptom-specific target with a neuronavigation system. However, both high-resolution structural MRI and neuronavigation systems are expensive and time consuming. Furthermore, these two equipment are currently not routinely used in most hospitals. Therefore, a simple approach that can fast and economically locate the two coordinates would greatly facilitate the application of these novel findings in clinical treatment.

For this purpose, we aimed to develop a scalp landmark-based approach to locate the coordinates given by Siddiqi et al., which requires only a few steps of measurements on the scalp. This approach was established in a group of adult subjects and tested for intra−/inter-rater reliability in an independent group. We further conducted a coil misplacement experiment to estimate the misplacement between the coil center and target during a 10-min stimulation without neuronavigation.

## Materials and methods

### Study design

**Figure 1:**
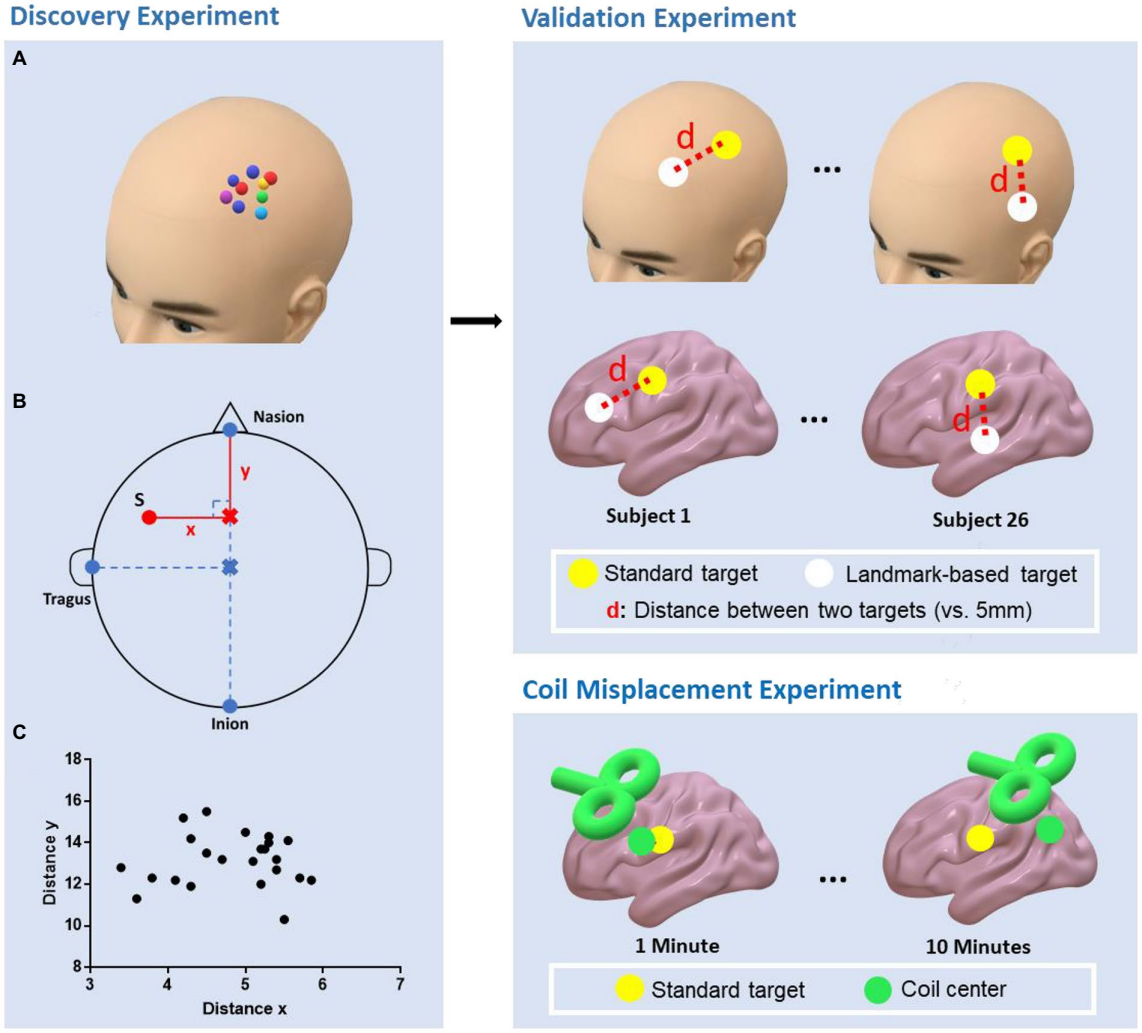
Study design of the experiments. Discovery experiment: The personalized targets were marked on the scalp using a neuronavigation system **(A)**; The parameters (distance *x*, distance *y*, distance_N-I_, and distance_V-T_) that were needed to determine the landmark-based approach were then measured. The personalized target was denoted as red point S **(B)**; The distribution of distance *x* and *y* **(C)**. Validation experiment: The “landmark-based approach” was replicated in an independent cohort and the personalized and landmark-based target distances to the TMS spatial resolution (i.e., 5 mm) were compared. Coil misplacement experiment: The misplacement between the coil center and target was estimated over 10 min of stimulation without neuronavigation.

The study consisted of three parts: (1) A discovery experiment to determine the parameters for locating the depression-related rTMS targets based on scalp landmarks. Siddiqi et al. (2020) reported three targets, two on the left frontal cortex and one on the right. The current study focused on the two targets on the left DLPFC. (2) a validation experiment to test the reliability and validity of the developed method from the discovery experiment and (3) a coil misplacement experiment to estimate the misplacement between the coil center and target during a 10-min stimulation without neuronavigation (Figure 1).

### Participants

Twenty-four subjects (ages 18–27, 12 females and 12 males, six were diagnosed with Major depression disorder according to DSM-V and 18 healthy subjects) were recruited for the discovery experiment and 26 (ages 17–45, 16 females and 10 males, six individuals with MDD and 20 healthy subjects) in the validation cohort. Two subjects from the validation cohort participated in the coil misplacement experiment. This study was approved by the local ethics committee and all subjects provided their written informed consent.

#### Discovery experiment

In the discovery experiment, the subjects were seated in a comfortable chair, wearing a fitting swimming cap. First, the two regions of interest centered at the respective MNI coordinates (anxiosomatic target [−37, 22, 54], dysphoric target [−32, 44, 34], radius = 6 mm) were transformed into each subject’s individual space by *SPM12* and *TMS target* software (Ji et al., 2017). Next, we used a neuronavigation system (Brainsight; Rogue Research, Montreal, Canada) to visualize individualized targets and marked the areas on the scalp. To facilitate the description, hereafter, we call these targets as the personalized targets Finally, four geodesic distances (in cm; Figure 1) described below were measured by an experienced TMS therapist (practice time > 1,000 h) with a tape ruler:

1. Distance from the nasion to inion (*distance_N-I_*): The researcher marked the halfway point of this line on the subject’s scalp, denoted as the vertex;
2. Distance from the vertex to the left tragus *(distance_V-T_)*;
3. The perpendicular distance between the personalized targets to the sagittal line over the scalp (*distance x*);
4. Distance from the nasion to the perpendicular point of the personalized targets on the sagittal line on the scalp (*distance y*).

The distances were averaged across subjects. We computed the ratio of mean *distance x* to mean *distance_V-T_* as *r_x_*, and the ratio of mean y to mean *distance_N-I_* as *r_y_*.

We proposed a “landmark-based approach” to locate the two symptom-specific targets by measuring the individualized *distance_V-T_* and *distance_N-I_*, respectively, multiplied the two by the two ratios *r_x_* and *r_y_*.

#### Validation experiment

In the validation experiment, we verified the reliability and validity of the “landmark-based approach” used in the discovery experiment. For reliability, we repeated the procedures of the discovery experiment, and compared the parameters of the landmark-based approach between experiments (distance *x*, distance *y*, distance_N-I_, and distance_V-T_, *r_x_*, and *r_y_*). In this experiment, each subject was estimated by two raters, twice per rater. Coordinates of the landmark-based targets in the Brainsight system were correlated between and within raters to show the reliability.

For validity, we compared the mean Euclidean distance between the personalized and landmark-based targets to the TMS spatial resolution. We use 5 mm as the TMS spatial resolution reported in other studies (Deng et al., 2013; Trapp et al., 2019). Distances larger than 5 mm indicated that the landmark-based approach navigated the stimulation to an unexpected brain structure.

#### Coil misplacement experiment

To examine the possible errors when conducting TMS sessions without neuronavigation, we next designed the coil misplacement experiment. There were two parts to this experiment, which tested the initial location and dynamic misplacement of the coil, respectively. The two raters of the validation experiment conducted the coil misplacement experiment. The anxiosomatic target was used as the personalized target. TMS was performed using a Magstim Rapid2 stimulator (Magstim Company) with a 70-mm air-cooled figure-8 coil.

The initial misplacement was the error when placing the coil on the scalp without neuronavigation. Specifically, the figure-8 TMS coil was placed on the marked personalized targets on the scalp without neuronavigation. Then, the initial coil misplacement was automatically determined by the Brainsight system. This step was conducted 15 times by two raters on each subject, with a total of 60 times for placement.

The dynamic misplacement was the coil misplacement during the rTMS session without neuronavigation. First, the TMS coil was placed on the personalized target with neuronavigation. Then, a train of 10-min sham rTMS was applied on the anxiosomatic target. The Brainsight system was then used to record the dynamic coil movement during the whole rTMS session. The raters were not allowed to check the neuronavigation interface during stimulation. The coil misplacement was recorded every 30 s. Each rater completed six rTMS sessions on each subject with at least 24 h intervals between each session for a total of 24 sessions.

### MRI acquisition

MRI data were collected at the University of Science and Technology of China (Hefei, Anhui Province) with a 3.0 T scanner (Discovery 750; GE Healthcare, Milwaukee, WI, USA). High-resolution anatomical images were acquired in the sagittal orientation using a three-dimensional brain-volume sequence (repetition/echo time, 8.16/3.18 ms; flip angle, 12; field of view, 256 mm × 256 mm; 256 × 256 matrix; section thickness, 1 mm, without intersection gap; voxel size, 1 mm × 1 mm × 1 mm; 188 sections) for later neuronavigation.

### Statistical analyses

In the discovery experiment, the mean values of distance *x*, distance *y*, distance_V-T_, and distance_N-I_ were calculated to determine the “landmark-based approach.”

In the validation experiment, for reliability, the Pearson’s correlation of the Brainsight coordinates of the landmark-based targets in three dimensions were calculated to test the inter-rater and intra-rater reliability. Spearman correlation coefficient was used if the data did not fit the normal distribution. The difference of distance *x* and *y* and *distance_V-T_* and *distance_N-I_* between the discovery and validation cohorts was compared using independent-samples *t*-tests. Validity was tested by comparing the TMS spatial resolution (i.e., 5 mm) and the distance between the personalized and landmark-based targets.

In the coil misplacement experiment, the distance between coil center and the target was compared to the TMS spatial resolution (i.e.,5 mm) using one-sample *t*-tests. The difference of initial/dynamic misplacement between subjects/raters was compared using independent samples *t*-tests.

## Results

### Discovery experiment

**Figure 2:**
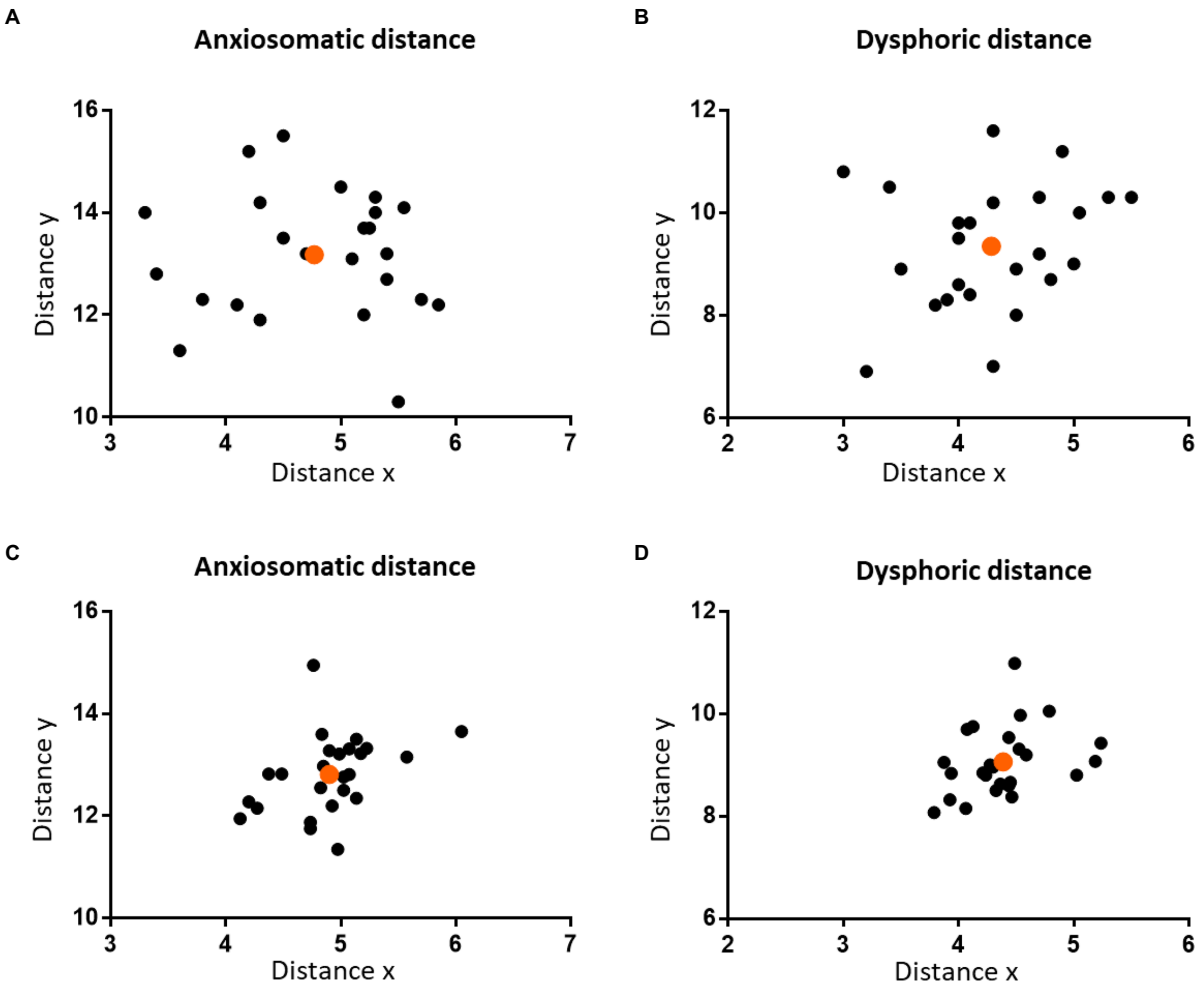
The distribution of the personalized targets on the scalp. The two figures in the upper panel show the distribution of the personalized anxiosomatic targets **(A)** and personalized dysphoric targets **(B)** on the scalp in discovery cohort, respectively. The two figures on the lower panel show the distribution of the personalized anxiosomatic targets **(C)** and personalized dysphoric targets **(D)** in the validation cohort, respectively. The mean position of each cohort is marked in orange.

The mean distance_V-T_ was 19.53 cm (SD = 0.62 cm) and mean distance_N-I_ was 36.19 cm (SD = 1.82 cm). For the anxiosomatic target, the mean distance ‘*x*’ was 4.77 cm (SD = 0.65 cm) and mean ‘*y*’ 13.18 cm (SD = 1.23 cm; Figure 2A). The *r_x_* and *r_y_* were 0.24 (95% confidence interval: 0.2282–0.2618) and 0.36 (95% confidence interval: 0.3511–0.3772), respectively. For the dysphoric target, the mean distance ‘*x*’ was 4.29 cm (SD = 0.75 cm) and mean ‘*y*’ 9.35 cm (SD = 1.22 cm; Figure 2B). The *r_x_* and *r_y_* were 0.22 (95% confidence interval: 0.2059–0.2452) and 0.26 (95% confidence interval: 0.2452–0.2715), respectively.

### Validation experiment

One of the subject’s failed to finish the whole validation experiment; thus, data from 25 subjects were used in the analysis.

#### Reliability outcome

Coordinates of the landmark-based target were correlated between- and within-raters (Table 1). In the validation cohort, the mean distance_V-T_ was 19.28 cm (SD = 0.52 cm) and mean distance_N-I_ 34.84 cm (SD = 1.31 cm). There was no statistical difference between the discovery cohort and validation cohort in distance_V-T_ (*p* = 0.119, *t* = 1.59); however, the difference of distance_N-I_ was significant (*p* = 0.013, *t* = 2.6).

**Table 1:** The correlation of Brainsight coordinates between-and within-raters.

	Anxiosomatic			Dysphoric		
	*X*	*Y*	*Z*	*X*	*Y*	*Z*
intra-rater1	0.86	0.96	0.99	0.83	0.92	0.99
intra-rater2	0.73	0.90	0.98	0.82	0.77	0.98
inter-rater	0.95	0.87	0.99	0.93	0.85	0.99

Pearson’s correlation was used in this table. The intra-rater reliability was calculated between the two time of measurements. The mean value of the two time of measurements for each rater was used to calculate the inter-rater reliability.

For the anxiosomatic target, the mean distance ‘*x*’ was 4.9 cm (SD = 0.42 cm) and mean ‘*y*’ 12.81 cm (SD = 0.76 cm). There were no statistical differences between the discovery cohort and validation cohort in distance *x* (*p* = 0.791, *t* = 0.266) or distance *y* (*p* = 0.191, *t* = 1.326; Figure 2C). The *r_x_* and *r_y_* of the anxiomatic target were 0.25 and 0.37 in the validation cohort and 0.24 and 0.36 in the discovery cohort, respectively. The difference of *r_x_* and *r_y_* between the two cohorts resulted in a cumulative error of around 3.98 mm. This error was within the threshold we predefined TMS spatial resolution (i.e.,5 mm), indicating sufficient reliability.

For the dysphoric target, the mean distance ‘*x*’ was 4.39 cm (SD = 0.38 cm) and mean ‘y’ 9.07 cm (SD = 0.67 cm). There was no statistical difference between the discovery cohort and validation cohort in distance *x* (*p* = 0.881, *t* = 0.151) or distance *y* (*p* = 0.261, *t* = 1.137; Figure 2D). The *r_x_* and *r_y_* of the dysphoric target were 0.23 and 0.26 in the validation cohort and 0.22 and 0.26 in the discovery cohort, respectively. The difference of *r_x_* and *r_y_* between the two cohorts resulted in a cumulative error of approximately 1.9 mm, which was within the 5 mm range, indicating sufficient reliability.

#### Validity outcome

**Figure 3:**
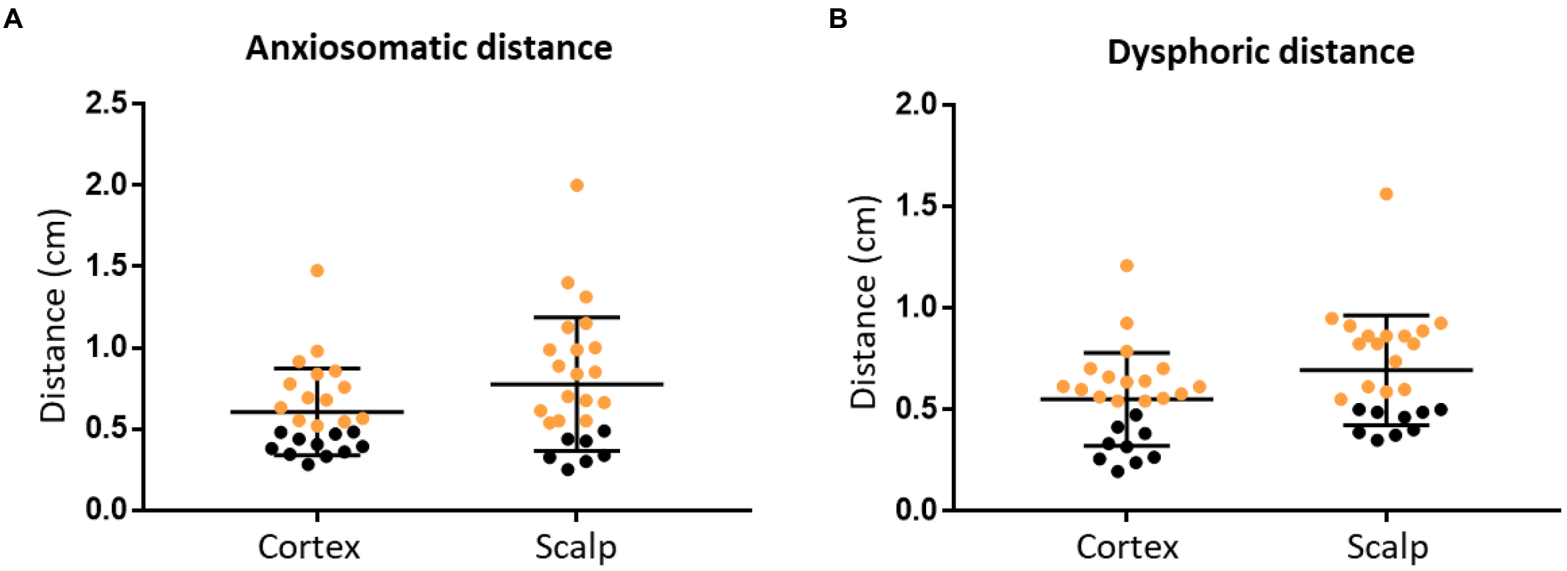
The personalized and landmark-based target distance of the two targets. Target distance of the anxiosomatic targets **(A)** and dysphoric targets **(B)**, respectively, on the cortex and scalp. The personalized and landmark-based target distances greater than 5 mm are marked in orange.

For anxiosomatic targets, there was no difference between the average personalized and landmark-based target distance (0.58 cm, SD = 0.21 cm) to 5 mm (*p* = 0.058, *t* = 1.988; Figure 3A). On the scalp, the average distance was 0.75 cm (SD = 0.35 cm). However, the personalized and landmark-based target distance in 52% of subjects (*n* = 13) was larger than 5 mm on the cortex.

For the dysphoric target, there was no difference between the average personalized and landmark-based target distance (0.54 cm, SD = 0.23 cm) to 5 mm (*p* = 0.286, *t* = 1.091; Figure 3B). On the scalp, the average distance was 0.68 cm (SD = 0.19 cm; Table 2). The target distance in 64% of subjects (*n* = 16) was larger than 5 mm on the cortex.

**Table 2:** The distance (cm) between the personalized and landmark-based targets in the validation experiment.

	Anxiosomatic target		Dysphoric target	
	Distance on the scalp (cm)	Distance on the cortex (cm)	Distance on the scalp (cm)	Distance on the cortex (cm)
Number of values	25	25	25	25
Minimum	0.25	0.28	0.35	0.2
25–75% Percentile	0.46–0.99	0.4–0.77	0.49–0.86	0.36–0.65
Median	0.68	0.55	0.61	0.56
Maximum	1.4	0.98	1.21	0.93
Mean	0.75	0.58	0.68	0.54
Std. Deviation	0.35	0.21	0.23	0.19

#### Coil misplacement outcome

##### Initial misplacement outcome

**Figure 4:**
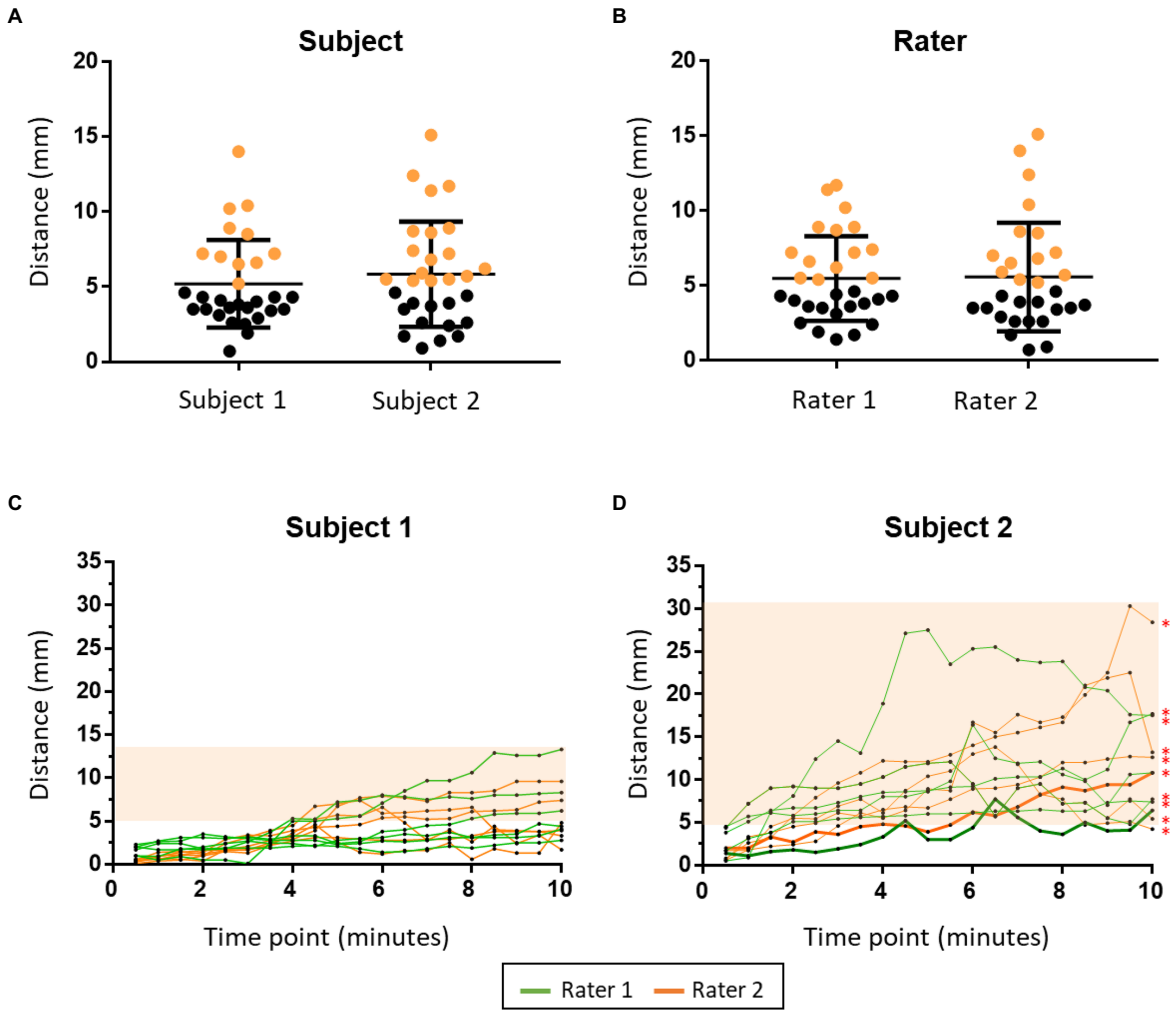
The findings of the coil misplacement experiment. The two figures in the upper panel show initial coil misplacement between subjects **(A)** and raters **(B)**. The misplacements greater than 5 mm are marked in orange. The two figures in the lower panel show the dynamic coil misplacement during the TMS session in subject 1 **(C)** and subject 2 **(D)**. The orange area shows the time points that have a dynamic coil misplacement larger than 5 mm. Thick lines in **(D)** show the rTMS session in which the mean dynamic coil misplacement was not significantly larger than 5 mm. ^*^: TMS session that has a mean dynamic coil misplacement significantly larger than 5 mm.

Each rater conducted 15 times of repeat coil placement on each subject (60 times in total). As compared with TMS spatial resolution (i.e., 5 mm), the mean value (5.52 mm, SD = 3.22 mm) of initial coil misplacement was not significant at the group level (*p* = 0.219, *t* = 1.243); however, at the individual level, misplacement of the coil greater than 5 mm occurred 47% (*n* = 28) of the time. There was no difference in initial coil misplacement (Between subjects: *p* = 0.446, *t* = 0.767; between raters: *p* = 0.906, *t* = 0.119) between subject 1 (5.2 mm, SD = 2.93 mm) and subject 2 (5.84 mm, SD = 3.51 mm) or between rater 1 (5.47 mm, SD = 2.83 mm) and rater 2 (5.57 mm, SD = 3.62 mm; Figure 4).

#### Dynamic misplacement outcome

Each subject received 12 rTMS sessions (six times from each rater, 24 times in total). There was a significant difference between the dynamic coil misplacement at all-time points (6.31 mm, SD = 5.14 mm) to 5 mm (*p* < 0.0001, *t* = 5.566). There was also a significant difference of mean dynamic coil misplacement (p < 0.0001, *t* = 12.68) between subject 1 (3.73 mm, SD = 2.59 mm) and subject 2 (8.88 mm, SD = 5.74 mm). The larger misplacement of subject 2 was caused by the unconscious head movement during stimulation. There was no difference of mean dynamic coil misplacement (*p* = 0.688, *t* = 0.402) between rater 1 (6.4 mm, SD = 5.26 mm) and rater 2 (6.21 mm, SD = 5.03 mm; Figure 4).

## Discussion

This study presented a fast and economical approach to locate two rTMS treatment targets for depression patients on the scalp that do not require MRI scan nor neuronavigation. This approach proved to have acceptable reliability and validity at the group level. However, the personalized and landmark-based target distances exceeded 5 mm in more than 50% of subjects. During the 10-min sham rTMS session, the average coil misplacement was significantly larger than 5 mm.

Both the inter-rater and intra-rater correlations were found to be significant in all three dimensions of the coordinates, suggesting a good reliability of the “landmark-based approach” developed in the current study. The personalized and landmark-based target distance results from our approach (anxiosomatic target: 5.8 mm, dysphoric target: 5.4 mm) were smaller than other scalp-based measurement methods (a mean distance of 8.31 mm; Weiduschat et al., 2009). But the accuracy of the current approach cannot compare with the accuracy of the neuronavigation system (mean distance 2.5 mm; Schönfeldt-Lecuona et al., 2005).

The validity analysis indicated that the “landmark-based approach” had acceptable validity at the group level. However, at the individual level, the variability of the landmark-based targets may be larger than observed, since the personalized and landmark-based target distances were larger than 5 mm in approximately 50% of the validation cohort. Larger sample sizes are needed to test whether this method has sufficient validity at the individual-level.

In addition to errors caused by the method itself, errors can occur during the TMS session without neuronavigation. Overall, 45% of the repeated placements of the TMS coil resulted in an initial coil misplacement larger than 5 mm. The mean dynamic coil misplacement at 10 min was 6.36 mm. A recent study found a relationship between TMS coil misplacement and the change of the TMS-induced electric field over time (Richter et al., 2013). According to the authors, when the coil misplacement was 6.36 mm, the relative decrease of the electric field was less than 8.4% compared at baseline. There is no denying that as the session time increases, the distance between the TMS coil and the target will gradually increase. Furthermore, the dynamic coil misplacement during the TMS session was found to be significantly different between the two subjects, indicating that some subjects likely had more pronounced head movements than others. For example, patients with mania, autism spectrum disorder (ASD) or transient tic disorder often find it difficult to restrict head movement. Therefore, for these types of patients, online neuronavigation is the optimal targeting approach.

In comparison with neuronavigation system and other locating methods that need individual brain imaging, the current method may have some advantages. This approach does not require to any expensive equipment like neuronavigation system or MRI scanner. This can benefit the hospitals that do not have access to these equipment. In addition, it helps to cut the economic and time cost of TMS treatment which is a common concern of clinical patients. For patients with MRI contraindication, this provides an alternative way to locate their stimulate site.

## Limitations

Our study has some limitations. First, most of our subjects from both cohorts were young, with an age range of 17 to 28 years; only one subject was older (47 years old). Future studies will likely need to conduct the method in different age groups, especially in older subjects and those with cerebral atrophy. Second, although there was no significant difference between the average personalized and landmark-based target distances to the TMS spatial resolution (i.e.,5 mm), the clinical efficiency still needs to be proven in practice. To further evaluate the actual effectiveness of this method, it will be necessary to conduct placebo-controlled trials. Third, the DLPFC was the personalized target in the coil misplacement experiment; it is undetermined whether this approach could be generalized to brain areas other than the DLPFC. Last, the current study was conducted by manual measurement, thus cannot avoid manual errors that may affect the stability and accuracy of the method.

Future studies can try to do the measurement completely on brain images with more state-of-the-art segmentation procedures and head models. This “*in silico*” approach will greatly help the researchers to collect data from larger samples and thus develop a more accurate method for target localization.

## Conclusion

The “landmark-based approach” is a fast and economical approach to locate two rTMS treatment targets for depression patients on the scalp that do not require MRI scan nor neuronavigation. The “landmark-based approach” can conveniently and reliably locate the two symptom-specific targets for depressive patients at group level. However, the accuracy is highly varied at individual level and needs to be further improved.

## Data availability statement

The raw data supporting the conclusions of this article will be made available by the authors, without undue reservation.

## Ethics statement

The studies involving human participants were reviewed and approved by the Medical Ethics Committee of Anhui Medical University. The patients/participants provided their written informed consent to participate in this study.

## Author contributions

RD: conceptualization, methodology, investigation, data curation, data acquisition, visualization, formal analysis, validation, and writing—original draft. QZ: data curation, data acquisition, investigation, and formal analysis. TH: data curation and investigation. JS and QH: investigation and validation. YW and YZ: investigation. KH: funding acquisition and resources. YT: funding acquisition. GJ: conceptualization, funding acquisition, formal analysis, supervision, and writing—review and editing. KW: conceptualization, funding acquisition, and resources. All authors contributed to the article and approved the submitted version.

## Funding

This study was funded by the National Natural Science Foundation of China, Grant/Award Numbers: 81971689 (GJ), 91432301 (KW), 32071054 (YT), 31571149 (KW), and 31970979 (KW); the Science Fund for Distinguished Young Scholars of Anhui Province, Grant Number: 1808085J23; Scientific Research Fund of Anhui Medical University: 2019xkj199; and the Collaborative Innovation Center of Neuropsychiatric Disorders and Mental Health of Anhui Province: 2020xkjT057.

## Conflict of interest

The authors declare that the research was conducted in the absence of any commercial or financial relationships that could be construed as a potential conflict of interest.

## Publisher’s note

All claims expressed in this article are solely those of the authors and do not necessarily represent those of their affiliated organizations, or those of the publisher, the editors and the reviewers. Any product that may be evaluated in this article, or claim that may be made by its manufacturer, is not guaranteed or endorsed by the publisher.
